# An Impassable Artificial Intestinal Valve during Double-Balloon Enteroscopy for Recurrent Cholangitis 18 Years after Choledochal Cyst Excision

**DOI:** 10.1055/a-2979-4687

**Published:** 2026-10-22

**Authors:** Yuka Kumata, Tetsuhiro Okada, Minori Ishii, Saya Azuma, Yuki Meya, Hisayuki Miyagi

**Affiliations:** 1 Division of Pediatric SurgeryDepartment of SurgeryAsahikawa Medical UniversityAsahikawaHokkaidoJapan; 2 Division of GastroenterologyDepartment of Internal MedicineAsahikawa Medical UniversityAsahikawaHokkaidoJapan

**Keywords:** choledochal cyst, double-balloon enteroscopy, artificial intestinal valve, cholangitis, transition care

## Abstract

Congenital biliary dilatation (formerly referred to as choledochal cyst) is a congenital anomaly characterized by cystic dilatation of the bile ducts. Historically, an artificial intestinal valve (AIV) was sometimes added to Roux-en-Y reconstruction to prevent postoperative reflux cholangitis, but its clinical significance remains controversial. We report the case of an 18-year-old man who underwent excision of congenital biliary dilatation and Roux-en-Y hepaticojejunostomy with an AIV during infancy. He developed recurrent cholangitis from 16 years of age. Magnetic resonance cholangiopancreatography showed mild intrahepatic bile duct dilatation and a microabscess in segment 5. Double-balloon enteroscopy-assisted endoscopic retrograde cholangiopancreatography was performed for biliary evaluation. However, the endoscope could not pass through the severely narrowed orifice of the AIV, which meant that reaching the hepaticojejunostomy was impossible. His symptoms improved with conservative antibiotic treatment, and no further cholangitis has occurred. He subsequently transitioned to the adult Gastroenterology Department and undergoes annual imaging follow-up. While AIVs are introduced to prevent reflux, recent studies have suggested that postoperative cholangitis is multifactorial. In this case, the AIV became a critical obstacle to endoscopic access approximately 20 years postoperatively. With advancements in double-balloon enteroscopy-assisted endoscopic retrograde cholangiopancreatography, securing future endoscopic access is increasingly important. An AIV can hinder endoscopic biliary access after congenital biliary dilatation surgery, and our institution has since discontinued its use.

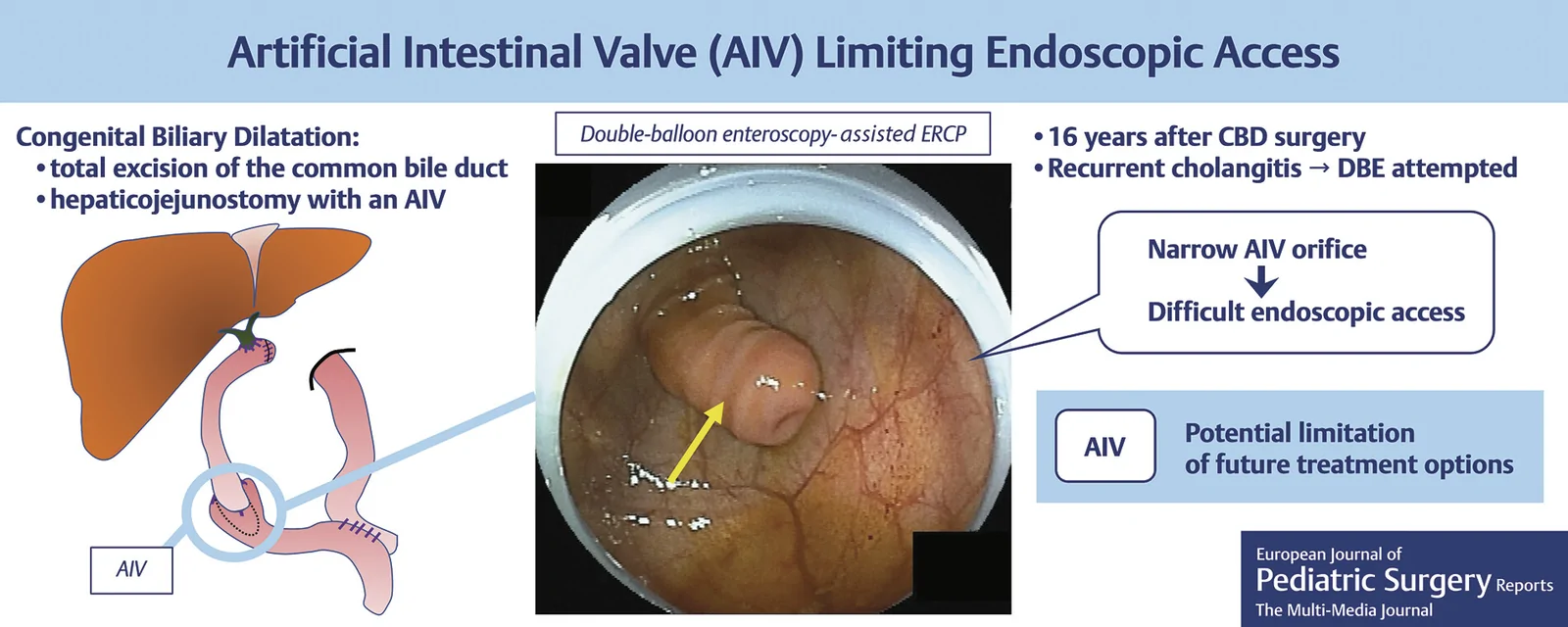

## Introduction

Congenital biliary dilatation ([CBD] choledochal cyst) is a congenital malformation closely associated with pancreaticobiliary maljunction and is characterized by cystic dilatation of the extrahepatic and/or intrahepatic bile ducts. The standard treatment for CBD is complete excision of the cyst and extrahepatic bile duct with bilioenteric reconstruction to separate the mutual reflux of bile and pancreatic juice. 1 Late postoperative complications affect 7 to 36% of patients, with cholangitis, biliary stricture, anastomotic stricture, and hepatobiliary malignancy reported as the most serious. 2 3 To prevent postoperative cholangitis, Nakajo et al introduced an intussusception-type artificial intestinal valve (AIV) at Roux-en-Y anastomosis in 1982. 4 Our institution introduced this AIV in 1991. However, we recently experienced a case in which an AIV made endoscopic access to the hepaticojejunostomy impossible in a patient with recurrent cholangitis 18 years after surgery.

## Case Presentation

An 18-year-old man was referred to a previous hospital at 1 month of age with jaundice, liver dysfunction, and a cystic lesion at the hepatic hilum. Suspecting CBD, he underwent total excision of the common bile duct and a retrocolic Roux-en-Y hepaticojejunostomy with an AIV at 56 days of life. Preoperative ultrasonography had shown cystic dilatation of the common bile duct, suggesting Todani type I or IV. The extrahepatic bile duct was dissected up to the normal-caliber hepatic duct, and the dilated portion was excised. The jejunum was divided 20 cm from the ligament of Treitz, and a Nakajo-style intussusception-type AIV was added 20 cm distal to the hepaticojejunostomy ( Fig. 1 ). The postoperative diagnosis was Todani type Ic. He was discharged without complications. At 5 years of age, he underwent radical surgery for a ventral hernia at the right subcostal incision. At 10 years of age, intrapancreatic remnant bile duct stones (protein plugs) were identified, for which he underwent excision of the remnant bile duct and stone extraction.

**Fig. 1:**
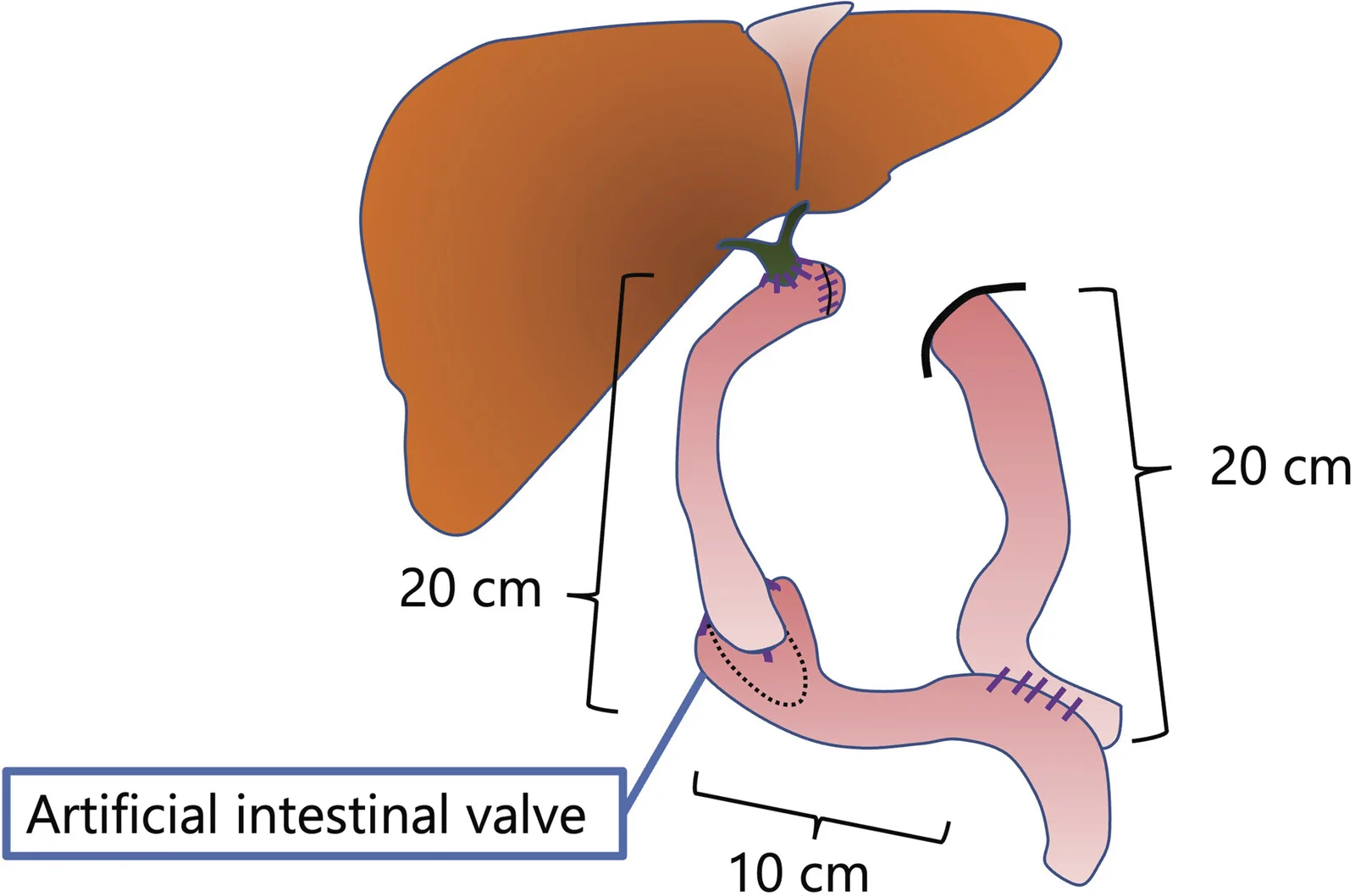
Schema of the biliary reconstruction. After total excision of the dilated common bile duct, a retrocolic Roux-en-Y hepaticojejunostomy was performed. A Nakajo-style artificial intestinal valve was created 20 cm distal to the anastomosis.

He presented with abdominal pain and fever at 16 and 17 years of age. On both occasions, laboratory tests showed leukocytosis but normal C-reactive protein, bilirubin, hepatobiliary enzyme, and amylase concentrations. Noncontrast computed tomography showed mild B5 intrahepatic bile duct dilatation without Roux limb dilatation, which was a new finding compared with computed tomography results 4 years earlier. No other source of fever was identified. These findings met the Tokyo Guidelines 2018 5 criteria for suspected, but not definite, acute cholangitis. At 16 years, he received cefmetazole for 5 days. At 17 years of age, two sets of blood cultures showed *Klebsiella pneumoniae* , and he received ampicillin/sulbactam for 2 days, cefmetazole for 3 days, and oral cefaclor for 1 week. Both episodes resolved with antibiotic therapy. At 18 years of age, he presented to the Emergency Department with fever and abdominal pain. Blood tests showed an elevated white blood cell count (17,880/μL) and C-reactive protein concentration (2.66 mg/dL), but bilirubin, hepatobiliary enzymes, and amylase concentrations were normal, as in the previous episodes. Abdominal ultrasonography and computed tomography showed localized dilatation of the B5 intrahepatic bile duct, similar in degree to the previous episodes, without stones, debris, or dilatation of the Roux limb. Magnetic resonance cholangiopancreatography, which was performed only during this episode, confirmed these findings and additionally identified a microabscess in segment 5 ( Fig. 2 ). Similar to the previous episodes, the findings met the Tokyo Guidelines 2018 criteria for suspected acute cholangitis. Under the suspicion of an anastomotic stricture as a potential cause, double-balloon enteroscopy-assisted endoscopic retrograde cholangiopancreatography was attempted but was aborted owing to difficulty with sedation. Therefore, he was managed conservatively with intravenous cefmetazole. Blood cultures remained negative, and his symptoms improved after 5 days of treatment. He was discharged on oral cefaclor for an additional 7 days, after which he had completed the course of antibiotic therapy.

**Fig. 2:**
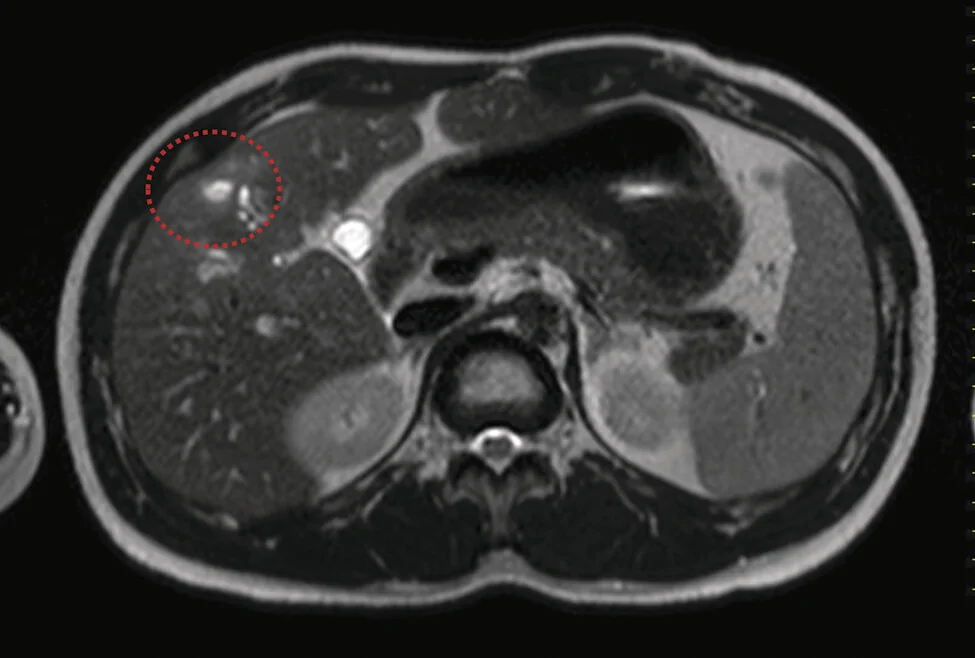
Magnetic resonance cholangiopancreatography. A microabscess (red circle) was identified in segment 5 of the liver.

Six months later, double-balloon enteroscopy-assisted endoscopic retrograde cholangiopancreatography was performed under general anesthesia for further evaluation. The endoscope was successfully inserted into the afferent limb, and the AIV was identified. However, the orifice of the valve was extremely small, which caused difficulty for the endoscope to pass, and reaching the hepaticojejunostomy was considered impossible ( Fig. 3A, B ). Fortunately, he has had no recurrence of cholangitis, and abdominal ultrasonography at 24 years of age confirmed resolution of the intrahepatic bile duct dilatation. He has transitioned to the adult Gastroenterology Department, where he is reviewed every 6 months and undergoes annual ultrasonography.

**Fig. 3:**
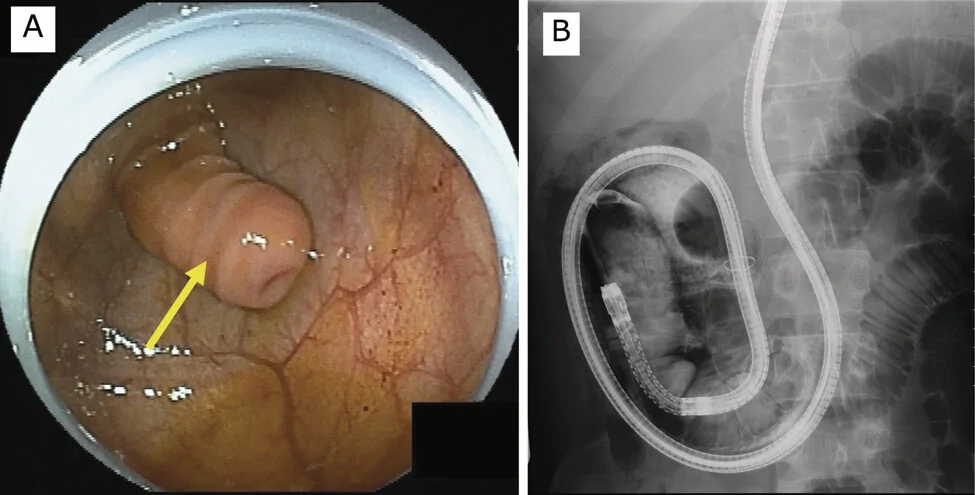
Double-balloon enteroscopy. ( **A** ) Photograph of double-balloon enteroscopy showing a severely narrowed orifice of the artificial intestinal valve (yellow arrow). ( **B** ) Fluoroscopic image of the double-balloon enteroscopy procedure.

## Discussion

AIVs have been added to Roux-en-Y reconstructions to prevent the reflux of intestinal contents into the afferent limb, aiming to prevent reflux cholangitis. However, the efficacy of AIVs remains controversial. In a single-center, prospective study of patients with biliary atresia, Ogasawara et al reported no significant difference in the incidence or frequency of postoperative cholangitis with or without an AIV, 6 although the applicability of these findings to CBD remains unclear. Furthermore, postoperative cholangitis is currently considered multifactorial, involving not only simple reflux but also biliary debris, stones, protein plugs, 7 biliary stasis, anastomotic stenosis, residual intrahepatic bile duct dilatation, 8 abnormal limb motility, 9 and an aberrant right hepatic artery coursing anteriorly. 10 These findings suggest that preventing reflux alone by an AIV may be insufficient to suppress cholangitis. Additionally, the valve itself has been reported to act as a starting point for bowel obstruction. 11

Two aspects of the AIV deserve consideration in this case. First, the AIV was unlikely to have been the primary cause of the recurrent cholangitis, because the biliary dilatation was confined to the B5 duct, and no Roux limb dilatation was observed in any imaging study. Second, and more importantly, the AIV substantially restricted future endoscopic access. Late complications after CBD surgery include cholangitis (7.5–14%), 3 12 intrahepatic stones (2.7–9%), 2 3 13 and biliary malignancy (0.7–5.4%). 14 15 Recently, advancements in double-balloon enteroscopy-assisted endoscopic retrograde cholangiopancreatography have enabled minimally invasive treatments, such as biliary evaluation, balloon dilatation of intrahepatic strictures, and lithotripsy, even in patients with a history of CBD surgery. 16 However, in this case, the AIV prevented the endoscope from reaching the bilioenteric anastomosis, abolishing the possibility of future endoscopic evaluation or intervention. As a result, a hilar or B5 duct stenosis could not be excluded because magnetic resonance cholangiopancreatography was not performed when there were no episodes of cholangitis. Nevertheless, the spontaneous resolution of the B5 dilatation by 24 years of age does not support the presence of a fixed stenosis. If cholangitis recurs, percutaneous transhepatic biliary drainage will be considered first, and if recurrences are frequent, resection of the AIV and reanastomosis may become a therapeutic option.

Published reports of AIV creation for CBD are limited to the 1980s and 1990s. 4 17 In our PubMed search, we identified no reports of AIV creation for CBD published after 1996. With modern advancements in endoscopic devices, securing future biliary access has become increasingly important. Therefore, biliary reconstruction in CBD surgery should be evaluated not only for short-term reflux prevention but also for enabling minimally invasive interventions in adulthood.

Long-term follow-up is essential for patients with CBD because late complications may appear more than a decade postoperatively. 2 Establishing a transition care system involving pediatric surgeons and adult gastroenterologists is crucial. In this case, the patient transitioned to adult gastroenterology at 24 years of age and continues to undergo regular surveillance.

## Conclusion

We experienced a case in which an AIV added during CBD reconstruction hindered endoscopic biliary access for cholangitis that developed 18 years postoperatively. On the basis of the potential need for future minimally invasive interventions for late complications, our institution has discontinued the use of AIVs following this experience.

## Notes

Importance for the Pediatric Surgeon We report a case in which an artificial intestinal valve created during childhood congenital biliary dilatation surgery completely prevented endoscopic access to the hepaticojejunostomy in adulthood. This finding indicates that pediatric surgeons should reconsider the use of artificial intestinal valves, prioritizing the preservation of long-term endoscopic biliary access over short-term reflux prevention, and recognize the importance of transition care.

## Declaration of GenAI Use

During the writing process of this paper, the author(s) used Claude (Anthropic) and ChatGPT (OpenAI) to assist with English–Japanese translation and to discuss the structure and logical flow of certain paragraphs. The authors reviewed and edited the resulting text and take full responsibility for the content of the paper.
